# A Time-Series Model of Phase Amplitude Cross Frequency Coupling and Comparison of Spectral Characteristics with Neural Data

**DOI:** 10.1155/2015/140837

**Published:** 2015-03-19

**Authors:** Kyle Q. Lepage, Sujith Vijayan

**Affiliations:** The Department of Mathematics & Statistics, Boston University, 111 Cummington Mall, Boston, MA 02215, USA

## Abstract

Stochastic processes that exhibit cross-frequency coupling (CFC) are introduced. The ability of these processes to model observed CFC in neural recordings is investigated by comparison with published spectra. One of the proposed models, based on multiplying a pulsatile function of a low-frequency oscillation (*θ*) with an unobserved and high-frequency component, yields a process with a spectrum that is consistent with observation. Other models, such as those employing a biphasic pulsatile function of a low-frequency oscillation, are demonstrated to be less suitable. We introduce the full stochastic process time series model as a summation of three component weak-sense stationary (WSS) processes, namely, *θ*, *γ*, and *η*, with *η* a 1/*f* ^*α*^ noise process. The *γ* process is constructed as a product of a latent and unobserved high-frequency process *x* with a function of the lagged, low-frequency oscillatory component (*θ*). After demonstrating that the model process is WSS, an appropriate method of simulation is introduced based upon the WSS property. This work may be of interest to researchers seeking to connect inhibitory and excitatory dynamics directly to observation in a model that accounts for known temporal dependence or to researchers seeking to examine what can occur in a multiplicative time-domain CFC mechanism.

## 1. Introduction

Cross frequency coupling (CFC) is a statistical relation between the phase or amplitude of a low frequency and the phase or amplitude of a high frequency. In this work, focus is placed upon phase-amplitude CFC, which can be thought of as the correlation of the amplitude of a relatively high-frequency oscillation (*γ*) with the phase of a lower frequency oscillation (*θ*).

The rationale behind this proposal is based on the experimental observations that (i) relatively slow frequency oscillations tend to be coordinated over large regions of neural tissue, unlike higher frequency oscillations [1, 2], and (ii) oscillatory activity reflects changes in the excitability of neural tissue [3]. Hence, a lower frequency oscillation may provide time intervals in which high-frequency activity may occur and may consequently coordinate high-frequency oscillations that are spatially disparate.

Recently, the theoretical interest in phase-amplitude CFC has been reinforced. Recent observations of the phenomenon have occurred in varied species [4–6], brain regions [7–9], and states of vigilance [10]. Experimental studies have shown that the nature of phase-amplitude CFC can be altered by predictive cues and attentional demands; the phase of the low-frequency oscillation can be reset such that a stimulus of attentional interest arrives at the phase of maximal excitability [11]. Furthermore, phase-amplitude CFC has been implicated in learning and memory [6, 12], and the dynamics of phase-amplitude CFC have been shown to change over the course of a cognitive task [8].

In this work, in a fashion akin to that classically employed for parametrically modeling the spectra of time-series, stochastic time-series models are constructed which exhibit cross frequency coupling (see, e.g., [13–15]). Through simulation it is shown that these models can produce time-series that exhibit CFC similar to that exhibited in neural recordings. Some mathematical properties of these models are given, and some consequences are discussed.

## 2. Methodology

Stochastic processes exhibiting cross frequency coupling are introduced. The ability for these processes to model observed cross frequency coupling in neural recordings is investigated and mathematical properties of the new models are given. Investigations are conducted through the use of mathematical analysis and simulation.

### 2.1. Model Specification

The stochastic, or random, processes introduced in this work model CFC phenomena in the following way. For each recorded measurement a random variable is introduced; the measurement is modeled as a realization of this random variable. The collection of random variables comprises a discrete-time random process. The observed time-series is modeled as the corresponding collection of realizations of each of the random variables in the random, or stochastic, process. This is the standard setup in classical time-series analysis [13] and it includes the independent and identically distributed (IID) random sample as a special case.

Each of the random processes is characterized by all of the possible moments between the random variables; in this work consideration will be restricted to random processes whose joint distributions are Gaussian. In this situation, specification of the first two joint moments completely specifies the model.

Without restriction upon the time dependence of pair-wise correlation, the number of unique pair-wise correlations increases quadratically with every new observation (in a single trial). This is a more challenging regime to perform inference than is typically considered, as in this case the number of unknowns is growing rapidly with increasing observation length. Contemporary work deals with this issue by recording many trials, or by using models with other restrictions.

Here, as is often customary, the introduced models are weak-sense stationary (WSS) random processes [13–15]. The weak-sense stationary property implies that the correlation between any pair of random variables in the process does not depend upon absolute time, but, rather, only upon the difference in the times associated with the pair. It also implies that the mean of each of the random variables in the process is equal; thus the process mean is also independent of time. Gaussian weak-sense stationary random processes are amongst the simplest of time-series models and the number of pair-wise correlations for these processes is of the same order as the number of measurements.

In this work, the random processes exhibiting CFC are constructed from component WSS processes modeling *θ*-rhythm, *γ*-rhythm, background activity, and sensor noise. The mean of these processes is taken to be zero, consistent with randomly observed neural phenomena (nonevoked). Based upon observed spectra, the autocorrelation of the *θ*, *γ*, and noise components is specified in the Fourier domain. Based upon the discrete-time analog of the Wiener-Khintchine theorem, the autocorrelation of each of these component processes is obtained by inverse discrete-time Fourier transforming the specified spectra [13].

#### 2.1.1. The Component Processes

Let *t* be the integer-valued time-index of the length *n* WSS zero-mean random process *θ* with autocovariance sequence *r* ^(*θ*)^:

(1) rτ(θ)=Eθtθt+τ−EθtEθt+τ,=Eθtθt+τ.

Here *E*{*X*} denotes the expected value of the random variable *X*. Similarly, specify WSS zero-mean processes for the *γ* rhythm and noise, *η*, components of the model. That is,

(2) $$\begin{array}{c} r_{\tau }^{\left(\gamma \right)}=E\left\{\gamma_{t}\gamma_{t+\tau }\right\},\\ r_{\tau }^{\left(\eta \right)}=E\left\{\eta_{t}\eta_{t+\tau }\right\}. \end{array}$$

Further, specify the *θ* and *η* components as uncorrelated. Because both *θ* and *η* are also zero-mean, it follows that *E*{*θ*_*t*_ *η*_*t*′_} is equal to zero. These components are jointly Gaussian, uncorrelated, and hence they are independent. The components *θ* and *γ* are linked to model CFC. This linking and its consequence are discussed in Section 2.1.2. It remains to specify the autocovariance sequences *r* ^(*θ*)^, *r* ^(*γ*)^, and *r* ^(*η*)^. As described, this is accomplished by specificying their respective spectra, *S* ^(*θ*)^, *S* ^(*γ*)^, and *S* ^(*η*)^, and using the example relation obtained by applying the discrete-time analog to the Wiener-Khintchine theorem [13]:

(3) $$\begin{array}{c} r_{\tau }^{\left(\theta \right)}=\Delta \overset{f_{N}}{\underset{-f_{N}}{\int }}S_{\theta }\left(f\right)e^{i2\pi f\tau \Delta }df. \end{array}$$

**Figure 1:**
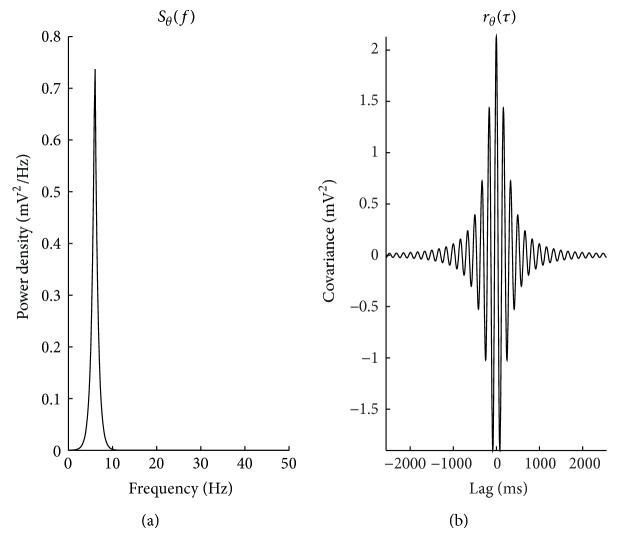
(a) *S*_*θ*_ specified as a 2 Hz peak exhibited in many neural recordings centered at 6 Hz. (b) The corresponding autocovariance sequence *r* ^(*θ*)^.

**Figure 2:**
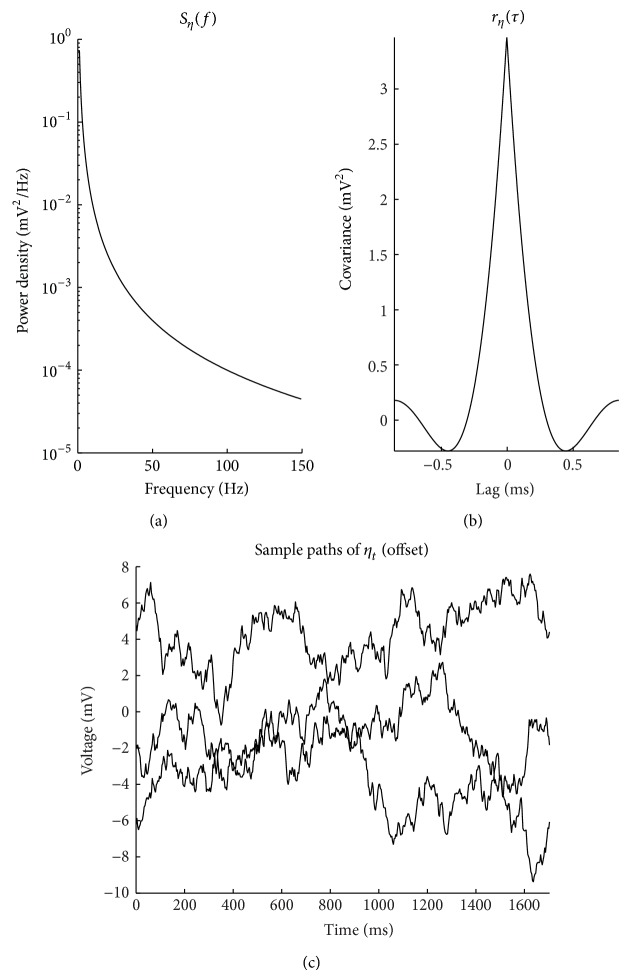
(a) *S*_*η*_ specified to be pink noise exhibiting the 1/*f* ^*α*^ decay with increasing frequency typically observed in neural recordings. (b) The corresponding autocovariance sequence *r* ^(*η*)^. Here *α* is equal to 2. (c) Sample paths of *η*.

Here *f*_*N*_ is the Nyquist frequency, equal to (2Δ)^−1^ (in Hz), specified in terms of the sample period Δ (in s). Figures 1 and 2 depict the specified model autocovariance sequences and spectra for the *θ* and *η* components (resp.). The *γ* component is further detailed in Section 2.1.2.

#### 2.1.2. Specification of *γ* Exhibiting Cross Frequency Coupling

In this work, CFC is specified by equating *γ* to a function *f*, relating *θ* to an unobserved, latent random process *x*. Let *x* be a zero-mean, WSS, random process possessing a spectrum *S*_*x*_, such that *S*_*x*_ is near zero outside of the frequency interval associated with a *γ*-rhythm. Candidate frequency intervals include low-*γ* (30 Hz–50 Hz) and high-*γ* (100 Hz–140 Hz). (These ranges can differ depending on the lab, species, and neural area. In [4], the low-*γ* frequency interval is specified to be 30 Hz–50 Hz, and the high-*γ* frequency interval is specified to be 80 Hz–150 Hz.) Consider

(4) $$\begin{array}{c} \gamma_{t}=f\left(\theta_{t},x_{t}\right), \end{array}$$

where ***θ***_*t*_ is a vector of random variables belonging to the *θ*-process. The *j*th element (***θ***_*t*_)_*j*_ of the random vector ***θ***_*t*_ is equal to *θ*_*t*+*j*_. The function *f* can be chosen to model various types of CFC. Together the process *x* and the function *f* control the dependence of *γ* upon *θ*. The relation (4) is general and can be used to model many types of CFC. For example, when

(5) $$\begin{array}{c} \gamma_{t}=\theta_{t+\tau }x_{t}, \end{array}$$

**Figure 3:**
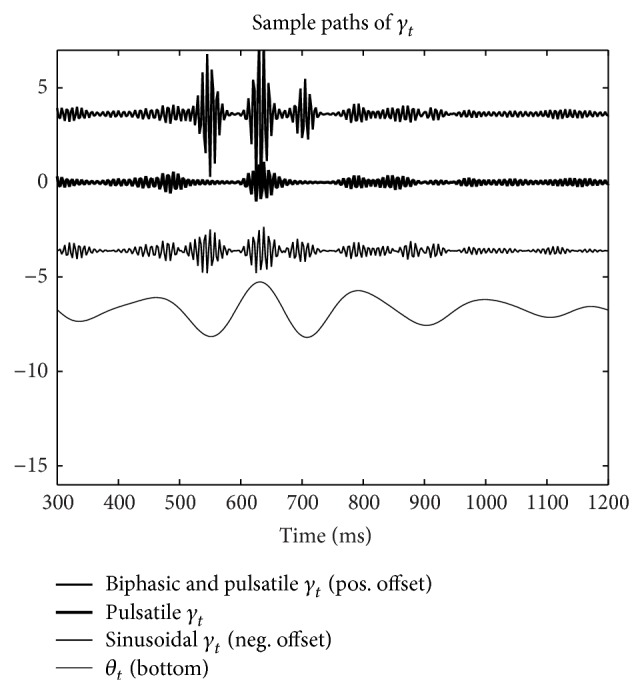
Sample paths of *γ* (scaled). Sinusoidal, pulsatile, and biphasic cross frequency coupling is exhibited. Each *γ* pulse is about 25 milliseconds in duration, consistent with a bandwidth of 40 Hz. These time-series approximate high-pass filtered neural recordings exhibiting CFC.

*γ* exhibits a phase shifted and sinusoidal variance (see Figure 3). When

(6) $$\begin{array}{c} \gamma_{t}=x_{t}\overset{K}{\underset{k=0}{\sum }}w_{k}\theta_{t+\tau }^{k}, \end{array}$$

*γ* exhibits a punctate interval of increased variance centered upon the prefered phase *ϕ*_*p*_, *ϕ*_*p*_ = *τ*/(2*πf*_*θ*_) (for the *K* equal to 4 case, with *w*_0_ = 0.4783, *w*_1_ = 0.2625, *w*_2_ = 0.0933, *w*_3_ = 0.0292, and *w*_4_ = 0.0058, see Figure 3). (To be pulsatile the Fourier transform of *γ* must have peaks spaced by *f*_0_. Here this is accomplished by summing *θ* taken to higher powers). When *θ*_*t*_ is squared in (6),

(7) $$\begin{array}{c} \gamma_{t}=x_{t}\overset{K}{\underset{k=0}{\sum }}w_{k}\theta_{t+\tau }^{2k}, \end{array}$$

and biphasic coupling is modeled (see Figure 3, *K* = 4, and *w*_*k*_ as specified in Figure 3). Note that doubling the power of *θ* in (7) doubles the harmonic frequency spacing present in the *γ* specified in (6). In the Appendix it is shown that the inverse tranform of a sequence with four harmonics in the frequency domain has a period equal to the inverse of the harmonic spacing.

#### 2.1.3. The Complete Time-Series Model

The model random process *y* generating the observed time-series is specified as the sum of the *θ*, *γ*, and *η* components. Consider

(8) $$\begin{array}{c} y_{t}=\theta_{t}+\gamma_{t}+\eta_{t}. \end{array}$$

### 2.2. Properties of the Model

Mathematical properties resulting from the specification (8) are explored by calculation.

#### 2.2.1. The Computation of *E*{*γ*_*t*_}

By definition the mean, *μ*_*t*_^(*γ*)^, of the *γ* component is

(9) μtγ=Eγt,=Eθt+τxt,=EθtτExt, independence  of  θ  and  x =0.

#### 2.2.2. The Computation of *E*{*y*_*t*_}

By definition, the process mean, *μ* ^(*y*)^, evaluated at time-index *t* is

(10) μt(y)=Eyt,=Eθt+γt+ηt,=0.

Since all three components *θ*, *γ*, and *η* are mean-zero processes. Thus, the model process *y* is also mean-zero.

#### 2.2.3. The Computation of *r*_*τ*_^(*γ*)^

By construction *θ*, *x*, and *η* are WSS random processes. To establish *γ* as a weak-sense stationary process, it is necessary to compute the autocovariance function *r* ^(*γ*)^. Begin with the definition for the general autocovariance function evaluated at the time indices *t* and *t* + *τ*. One has

(11) rt,t+τ′(γ)=Eγtγt+τ−EγtEγt+τ,=Eθt+τxtθt+τ+τ′xt+τ′.  

From an application of the Isserlis formula [14, 16], the following relation results:

(12) rt,t+τ′γ=Eθt+τxtθt+τ+τ′xt+τ′,=Eθt+τθt+τ+τ′Extxt+τ′,=rτ′θrτ′x,=rτ′γ,

which is independent of *t*.

#### 2.2.4. The Spectrum, *S*_*γ*_, of *γ*

The discrete-time Fourier transform of *r* ^(*γ*)^ is equal to *S*_*γ*_. From an application of the discrete-time convolution theorem, the following expression relating *S*_*θ*_ and *S*_*x*_ to *S*_*γ*_ is obtained:

(13) $$\begin{array}{c} S_{\gamma }\left(f\right)=\overset{f_{N}}{\underset{-f_{N}}{\int }}S_{\theta }\left(f^{\prime }\right)S_{x}\left(f-f^{\prime }\right)df^{\prime }. \end{array}$$

#### 2.2.5. Cross Frequency Coupling and the Frequency of *θ*

**Figure 4:**
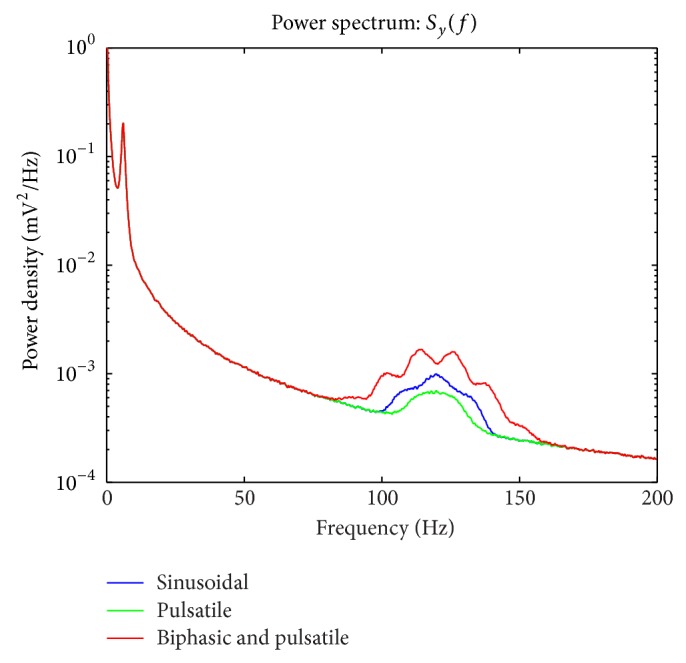
Spectra *S*_*y*_ of the stochastic model *y*. The sinusoidal, pulsatile, and biphasic *γ*'s each contribute to the spectrum *S*_*y*_ in a different way. The spectrum associated with *γ* computed with the pulsatile function of *θ* is similar to the spectrum estimated from actual neural recordings (see [5]). The spectra associated with sinuosoidal, pulsatile, and biphasic *γ* exhibit side-lobes consistent with the convolution appearing in (13), (21).

**Figure 5:**
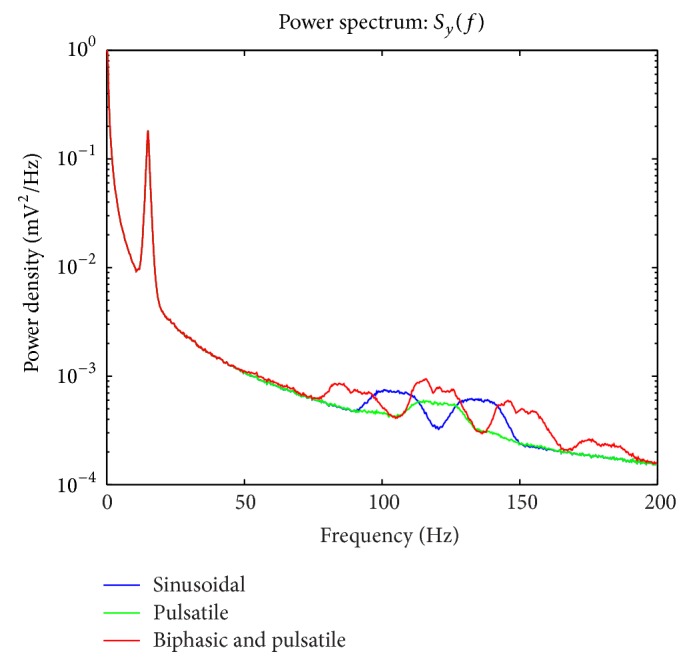
Spectra *S*_*y*_ of the stochastic model *y* with *θ* centered upon 15 Hz. The sinusoidal, pulsatile, and biphasic *γ*'s each contribute to the spectrum *S*_*y*_ in a different way.

To explore the effect of *θ* center frequency upon cross frequency coupling, in simulation, two center frequencies are used. These frequencies are 6 Hz (Figure 4) and 15 Hz (Figure 5). The effect of making this change can be seen by comparing the spectra plotted in Figure 4 with those plotted in Figure 5.

#### 2.2.6. The Effect of *γ* Bandwidth on Pulse Duration

Since *S*_*γ*_ is approximately nonzero only on a 40 Hz interval, the absolute-autocovariance, |*r*_*t*_^(*γ*)^|, is approximately nonzero for lags of 1/40 seconds. Thus, pulses will tend to have about a 25-millisecond duration.

#### 2.2.7. Simulated Time-Series: Sample Path Generation

To obtain a length*n* sample-path, that is, a truncated realization, of any one of the random processes *θ*, *x*, or *η* described in this work, a draw from *n* IID standard Gaussian random variables is made (mean-zero, unit variance). These Gaussian random variables can be collected into an *n*-dimensional vector **v**. The difference between **v** and the desired random vector **y**, with covariance matrix **R**, is

(14) $$\begin{array}{c} y=R^{1/2}v, \end{array}$$

since

(15) covy=EyyT−EyEyT,=EyyT,=R1/2EvvTR1/2,=R.

Thus, if **R** is known, then a realization of **y** can be computed by multiplying a realization of **v** by **R** ^1/2^. It remains to specify **R**. For a weak-sense stationary process, the covariance between random variables depends only upon their separation in time-index. Thus, **R** is Toeplitz with constant diagonals and is completely specified by the autocovariance function. For example, for the process *y*,

(16) $$\begin{array}{c} {\left(R^{\left(y\right)}\right)}_{t,t+\tau }=r_{\tau }^{\left(y\right)}. \end{array}$$

This method of computing simulated realizations is used for all processes in this work except for *γ*, and hence, for *y*. In the former case, a realization of *γ* is computed from realizations of *θ* and *x*. Similarly, a realization of *y* is computed by summing realizations of *θ*, *γ*, and *η*.

### 2.3. Model Assessment

The appropriateness of *y* as a model of actual recordings exhibiting CFC is assessed, somewhat crudely, by comparing visually the sample paths of *γ* generated with the three different *f* functions relating *θ* and *x* to *γ* and by comparing the spectrum of *y* and *γ* to that exhibited [5] in actual neural recordings.

## 3. Results

### 3.1. Simulated *γ* Time-Series Exhibiting CFC

Figure 3 depicts sample paths of *γ* constructed, using (5), (6), and (7). Here (5) corresponds to the label “Sinusoidal *γ*_*t*_,” (6) corresponds to the label “Pulsatile *γ*_*t*_,” and (7) corresponds to the label “Biphasic *γ*_*t*_.” These time-series approximate high-pass filtered neural recordings exhibiting CFC.

### 3.2. The Autocovariance Sequence *r*_*y*_

Having established that *y* is mean-zero, the autocovariance of *y* is equal to

(17) rt,t+τy=Eytyt+τ,=Eθt+γt+ηt×θt+τ+γt+τ+ηt+τ.

Due to independence and the fact that *E*{*η*_*t*_} is equal to zero, the following is considered:

(18) rt,t+τ(y)=Eθt+γtθt+τ+γt+τ+rτ(η),=rτ(θ)+rτ(γ)+Eθtγt+τ+Eγtθt+τ+rτ(η),

but

(19) Eθtγt+τ=Eθtθt+τxt+τ,=Eθt,θt+τExt+τ ∣ θt,θt+τ,=Eθt,θt+τExt+τ, (by  independence)=0.

Similarly, *E*{*γ*_*t*_ *θ*_*t*+*τ*_} is also zero. Then

(20) $$\begin{array}{c} r_{\tau }^{\left(y\right)}=r_{\tau }^{\left(\theta \right)}+r_{\tau }^{\left(\gamma \right)}+r_{\tau }^{\left(\eta \right)}. \end{array}$$

### 3.3. *y* Is Weak-Sense Stationary (WSS)

The three components *θ*, *γ*, and *η* are each mean-zero, and hence *y* is also mean-zero. Since the autocovariance *r* ^(*y*)^ is completely specified in (20) in terms of *r* ^(*θ*)^, *r* ^(*γ*)^, and *r* ^(*η*)^, each of which does not depend upon absolute time, *r* ^(*y*)^ also does not depend upon absolute time. Since the first two moments of the process *y* are independent of absolute time, *y* is WSS.

### 3.4. The Spectrum *S*_*y*_ of *y*

Applying (3) to (20) results in the following relation:

(21) Syf=Sθ(f)+Sγ(f)+Sη(f),=Sθf+Sηf+∫−1/21/2Sθf′Sxf−f′df′.

### 3.5. Time-Series Models of Cross Frequency Coupling (CFC)

Using the three *θ*, *x* coupling functions (sinusoidal, pulsatile, and biphasic pulsatile) used to specify *γ* and the simulation method discussed in Section 2.2.7, 5000 realizations of *y* are simulated. From each of these realizations, an estimate of *S*_*y*_ is computed. These estimates are averaged to obtain the three curves depicted in Figure 4. The convolution between *f*(*θ*) and *x* produces prominent side-lobes in the spectra of *y* for the sinusoidal and biphasic cases. For the pulsatile case the spectrum of *y* appears similar to those exhibited by actual neural recordings [5].

### 3.6. *S*_*y*_ for a Frequency-Shifted *θ*

**Figure 6:**
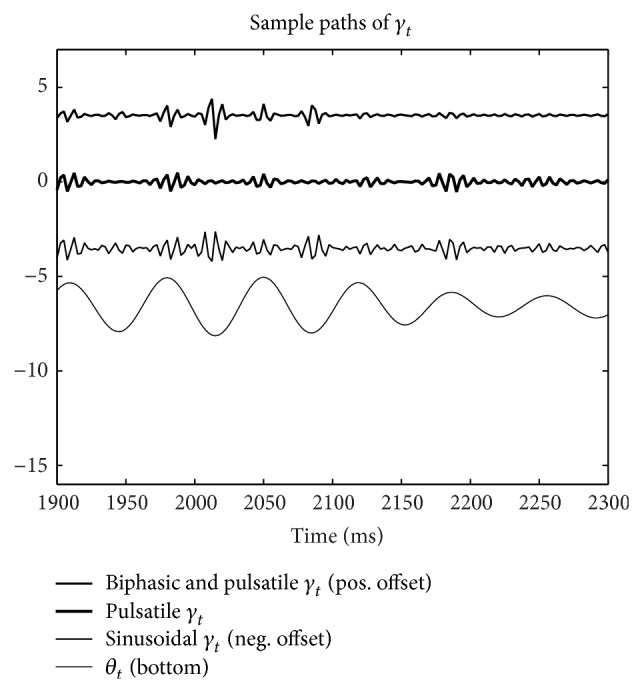
Sample paths of *γ* (scaled). In this case *γ* is coupled to a 15 Hz *θ*. For comparison with a *θ* centered about 6 Hz see Figure 3. Sinusoidal, pulsatile, and biphasic cross frequency coupling is exhibited. Each *γ* pulse is about 25 milliseconds in duration, consistent with a bandwidth of 40 Hz. These time-series approximate high-pass filtered neural recordings exhibiting CFC.

The simulation presented in Section 3.5 is repeated for a modified *θ*. Specifically, the frequency about which the *θ* component is centered is shifted from 6 Hz to 15 Hz. The resulting spectra associated with the sinusoidal, pulsatile, and biphasic *θ*-*γ* coupling are depicted in Figure 5. Sample paths of the associated *γ* are shown in Figure 6.

## 4. Discussion

The stochastic models considered in this work are weak-sense stationary. Phenomena without the WSS property, such as stimulus transients and time varying changes in autocovariance, are not captured by these models. While the topic is left for future study, one expects a convolution similar to that producing *γ* to appear in the nonstationary analogs of the time-series models considered in this work.

In [17] the probability density function for a phase-amplitude coupling estimator is provided under the assumption that phase-amplitude CFC is absent. The parametric regression based phase-amplitude coupling estimators [18, 19] are based upon conditional expectations which may not completely reflect the statistical properties of recordings that exhibit phase-amplitude coupling. The time-series models explicitly proposed in this work account for realistic temporal dependency and exhibit phase-amplitude CFC. As in [17], sampling properties of proposed estimators can be assessed based on a time-series model.

Owing to the convolution integral present in (13) and (21), the time-series introduced in this work exhibit, in general, multiple peaks about the 120 Hz center frequency of *γ*. This effect is minimized for CFC developed through the product of the latent process *x* with the pulsatile function *f*(*θ*) specified in (6). Of the considered models, it is this latter model which most closely matches observed CFC phenomena. The pulsatile function can be loosely thought of as imposing a window over each cycle of the slow oscillation during which the latent and relatively high-frequency process *x* is amplified. In the neural context, one might infer the existence of a similar window defining intervals of time when neural activity is more easily generated, consistent with the notion that oscillatory activity represents cyclic changes in excitability [3].

The excitability described in [3] is considered to be a function of the relative levels of synaptic inhibition and excitation. Pulsatile functions that more closely match the shape and time-course of synaptic currents may more closely match the observed features of phase-amplitude CFC in neural data. By comparing the simulated data resulting from the shape and time course of a simulated pulsatile function to actual neural data, insight may be gleaned regarding the nature of synaptic currents underlying phase-amplitude CFC. This modeling process, beginning with a stochastic time-series model of observed phenomena, may also provide insight into the other types of CFC, namely, amplitude-amplitude and phase-phase cross frequency coupling.

The process of time-series model simulation and the comparison of simulation results to results obtained from neural data can be facilitated with the use of statistical methods. The quantities in the proposed time-series models can be estimated, confidence intervals can be provided, model selection can be performed, and across-condition comparisons and hypothesis tests can be developed. Work refining these models and developing associated statistical methodology is underway.

The time-series models introduced in this work are, to the best of the authors' knowledge, the first reported time-series models capable of generating phase-amplitude cross frequency coupling. They may provide a basis for a more accurate and statistically principled assessment of cross frequency coupling and underlying synaptic activity that accounts for the temporal dependencies present in actual recordings.

Finally, the method of simulating sample-paths of weak-sense stationary processes introduced in Section 2.2.7 seems to be novel to the neuroscience community. Its use may facilitate increased accuracy when numerically investigating the behavior of statistics computed from neural recordings.

## 5. Conclusion

Full appreciation of the effect of different time-domain manifestations of cross frequency coupling may provide insight into neural processes. In particular, prominent spectral features are produced by somewhat mild changes to the nature of a time-series model exhibiting phase-amplitude cross frequency coupling. These changes may provide the basis for refined statistical methodology capable of shedding light into the nature of the inhibitory and excitatory synaptic currents supporting phase-amplitude cross frequency coupling.

## Appendix

### DTFT of a Periodic Sequence

Let *g* be a sequence with discrete-time Fourier transform (DTFT) *G*. Then

(A.1) $$\begin{array}{c} g_{t}=\overset{f_{N}}{\underset{-f_{N}}{\int }}G\left(f^{\prime }\right)e^{i2\pi f^{\prime }t\Delta }. \end{array}$$

Further, let *G*(*f*) be nonzero only for *f* = *n*/*τ*Δ, *n* = −4, −3,…, 3,4. Then

(A.2) gt=∫−fNfNGf′ei2πf′tΔ,=G(0)+∑n=−4n≠04GnτΔei2nπ(t/τ).

Because for each *n*, *e* ^*i*2*πn*(*t*/*τ*)^ is periodic with period *τ*, *g* is also periodic with period *τ*.

## References

[1] Dickson C. T., Biella G., De Curtis M. (2000). Evidence for spatial modules mediated by temporal synchronization of carbachol-induced gamma rhythm in medial entorhinal cortex. *The Journal of Neuroscience*.

[2] von Stein A., Sarnthein J. (2000). Different frequencies for different scales of cortical integration: from local gamma to long range alpha/theta synchronization. *International Journal of Psychophysiology*.

[3] Lakatos P., Shah A. S., Knuth K. H., Ulbert I., Karmos G., Schroeder C. E. (2005). An oscillatory hierarchy controlling neuronal excitability and stimulus processing in the auditory cortex. *Journal of Neurophysiology*.

[4] Canolty R. T., Edwards E., Dalal S. S. (2006). High gamma power is phase-locked to theta oscillations in human neocortex. *Science*.

[5] Scheffzük C., Kukushka V. I., Vyssotski A. L., Draguhn A., Tort A. B. L., Brankačk J. (2011). Selective coupling between theta phase and neocortical fast gamma oscillations during REM-sleep in mice. *PLoS ONE*.

[6] Tort A. B. L., Komorowski R. W., Manns J. R., Kopell N. J., Eichenbaum H. (2009). Theta–gamma coupling increases during the learning of item–context associations. *Proceedings of the National Academy of Sciences of the United States of America*.

[7] Chrobak J. J., Buzsáki G. (1998). Gamma oscillations in the entorhinal cortex of the freely behaving rat. *The Journal of Neuroscience*.

[8] Tort A. B. L., Kramer M. A., Thorn C. (2008). Dynamic cross-frequency couplings of local field potential oscillations in rat striatum and hippocampus during performance of a T-maze task. *Proceedings of the National Academy of Sciences of the United States of America*.

[9] Voytek B., Canolty R. T., Shestyuk A., Crone N. E., Parvizi J., Knight R. T. (2010). Shifts in gamma phase-amplitude coupling frequency from theta to alpha over posterior cortex during visual tasks. *Frontiers in Human Neuroscience*.

[10] Brankačk J., Scheffzük C., Kukushka V. I., Vyssotski A. L., Tort A. B. L., Draguhn A. (2012). Distinct features of fast oscillations in phasic and tonic rapid eye movement sleep. *Journal of Sleep Research*.

[11] Lakatos P., Karmos G., Mehta A. D., Ulbert I., Schroeder C. E. (2008). Entrainment of neuronal oscillations as a mechanism of attentional selection. *Science*.

[12] Lisman J. E., Jensen O. (2013). The *θ* − *γ* neural code. *Neuron*.

[13] Priestley M. B. (1981). *Spectral Analysis and Time Series*.

[14] Percival D. B., Walden A. T. (1993). *Spectral Analysis for Physical Applications*.

[15] Brillinger D. R. (2001). *Time Series: Data Analysis and Theory*.

[16] Isserlis L. (1918). On a formula for the product-moment coefficient of any order of a normal frequency distribution in any number of variables. *Biometrika*.

[17] Özkurt T. E. (2012). Statistically reliable and fast direct estimation of phase-amplitude cross-frequency coupling. *IEEE Transactions on Biomedical Engineering*.

[18] Penny W. D., Duzel E., Miller K. J., Ojemann J. G. (2008). Testing for nested oscillation. *Journal of Neuroscience Methods*.

[19] Kramer M. A., Eden U. T. (2013). Assessment of cross-frequency coupling with confidence using generalized linear models. *Journal of Neuroscience Methods*.

